# Effect of long-term intake of *α*-lactalbumin on menstruation-related symptoms and quality of life in healthy Japanese women: a randomized, double-blind, placebo-controlled study

**DOI:** 10.3389/fnut.2026.1892605

**Published:** 2026-07-24

**Authors:** Kentaro Ito, Kae Yamazaki, Anna Natori

**Affiliations:** Division of Research and Development, Meiji Co., Ltd., Hachioji, Tokyo, Japan

**Keywords:** alpha-lactalbumin, menstruation-related symptoms, premenstrual syndrome, quality of life, women’s health

## Abstract

**Background/objectives:**

Coping strategies for menstruation-related symptoms are required to support women’s health-related quality of life (QoL) and the continued social advancement of women. Previous studies have reported that short-term intake of *α*-lactalbumin (aLA) alleviates physical symptoms during menstruation. In this study, we investigated the effects of long-term intake of aLA on menstruation-related symptoms and QoL in healthy Japanese women.

**Methods:**

A randomized, double-blind, placebo-controlled study was conducted. Participants were 140 healthy women aged 20–39 years and were randomly assigned to receive aLA (900 mg/day) or placebo for three menstrual cycles. Premenstrual and intramenstrual symptoms were assessed at each cycle. The primary outcome was subjective symptoms on the Menstrual Distress Questionnaire (MDQ), and secondary outcomes were the 36-Item Short Form Health Survey (SF-36), subjective symptoms on the Visual Analog Scale, and urinary prostaglandins.

**Results:**

The primary outcome, the MDQ, showed no significant differences in the total score or the six factor scores between groups, either premenstrually or during menstruation. In exploratory analyses, aLA intake significantly alleviated the premenstrual MDQ sub-scores “Weight gain” and “Lowered motor coordination” compared with placebo (*p* < 0.05 for each). Among the secondary outcomes, aLA intake significantly improved the premenstrual role-related and social QoL scores on the SF-36 compared with placebo (*p* < 0.05). No significant group differences were observed for other items.

**Conclusion:**

In this trial, long-term aLA supplementation did not improve overall menstruation-related symptoms as assessed by the primary outcome. However, secondary outcomes indicated potential benefits in role-related and social aspects of premenstrual QoL. These findings are exploratory, requiring further studies for confirmation.

**Clinical trial registration:**

https://center6.umin.ac.jp/cgi-open-bin/ctr_e/ctr_view.cgi?recptno=R000063507, UMIN-CTR UMIN000055576

## 1 Introduction

Many women experience menstruation-related symptoms that markedly impair their quality of life (QoL) in health-related and social domains. Menstruation-related symptoms include premenstrual syndrome (PMS) and symptoms during menstruation, such as menstrual pain (dysmenorrhea, particularly when symptoms are severe). PMS is characterized by physical and psychological symptoms that occur in the luteal phase before menstruation and resolve with the onset of menstruation (1). When psychological symptoms predominate and are severe, the condition is diagnosed as premenstrual dysphoric disorder (PMDD).

Effective coping strategies for menstruation-related symptoms are essential for maintaining women’s QoL. International surveys have reported that the prevalence of PMS is approximately 24% (2). Regarding symptoms during menstruation, a survey of Dutch women reported that 85% experienced dysmenorrhea, 77% reported psychological complaints, 71% experienced fatigue, and 38% had difficulty performing daily activities during menstruation (3).

Although the pathophysiology of PMS has not been fully elucidated, dysfunction of the serotonin system is widely considered to play a central role (4, 5). Fluctuations in blood serotonin levels during the luteal phase have been observed in patients with PMS (6). Moreover, accumulating clinical evidence indicates that selective serotonin reuptake inhibitors (SSRIs) alleviate both psychological and physical symptoms of PMS (7, 8). Abnormalities in serotonergic function are closely associated with cyclical fluctuations in progesterone. Allopregnanolone, a progesterone metabolite, has been reported to affect serotonergic neural activity via *γ*-aminobutyric acid (GABA) receptors (9, 10). Rapid hormonal fluctuations during the luteal phase are therefore thought to destabilize the serotonergic system, leading to symptoms such as mood dysregulation and depression.

Menstrual pain is primarily caused by excessive production of prostaglandins (PGs) in the endometrium. The major PGs produced in the endometrium are prostaglandin F2α (PGF2α) and prostaglandin E2 (PGE2), both of which have been implicated in dysmenorrhea (11–13). During menstruation, the decline in progesterone associated with luteal regression increases cyclooxygenase-2 (COX-2) expression in the endometrium, thereby enhancing the synthesis of PGF2*α* and PGE2 (14). These PGs induce strong myometrial contractions, increase intrauterine pressure, and cause uterine ischemia, resulting in pain. When released into the systemic circulation, they may also induce symptoms such as headache, nausea, vomiting, and diarrhea (15).

α-Lactalbumin (aLA) is a whey protein that accounts for approximately 4% of total protein in bovine milk and has been reported to exert various health benefits. Clinical studies have suggested that aLA intake improves depressed mood (16), short-term memory (17), and sleepiness (18). These effects are thought to be attributable to the high tryptophan (Trp) content of aLA, since Trp serves as a precursor for serotonin synthesis. In addition, animal studies have reported that aLA has anti-inflammatory and analgesic effects (19), inhibits carcinogenesis (20), improves impaired glucose tolerance (21), and suppresses hepatitis (22, 23). These effects are thought to be mediated primarily through its anti-inflammatory action, via inhibition of COX-2 (19). Thus, aLA may exert beneficial effects on both psychological and physical health through its nutritional and pharmacological properties.

Several studies have suggested that aLA is effective against menstruation-related symptoms. For example, aLA has been suggested to relieve menstrual pain (24), alleviate physical symptoms during menstruation (25), and partially improve premenstrual memory function (26). However, these studies were limited by their short duration, typically involving only a single menstrual cycle or a single dose of aLA. Thus the effects of long-term aLA intake over multiple menstrual cycles remain unclear. Given the substantial intra-individual variability in menstruation-related symptoms (27, 28), evaluation of longer-term interventions is particularly important. Furthermore, it has not been examined whether the alleviation of symptoms by aLA leads to improvements in women’s QoL. Accordingly, the present study aimed to investigate the effects of long-term aLA intake over three consecutive menstrual cycles on menstruation-related symptoms and QoL in healthy Japanese women in a randomized, double-blind, placebo-controlled trial.

## 2 Materials and methods

### 2.1 Study design

This study was designed as a randomized, double-blind, placebo-controlled, parallel-group trial. The study protocol was developed in accordance with the Declaration of Helsinki and was approved by the Japan Conference of Clinical Research Institutional Review Board (approval date: September 26, 2024; approval number: 2403–028). Subsequently, an amendment to the protocol was submitted and approved to allow for minor changes, including an extension of the study period, changes in research staff due to transfer, and the correction of typographical errors (approval date: July 25, 2025).

The study protocol was registered in the UMIN Clinical Trials Registry before study initiation (registration date: September 27, 2024; ID: UMIN000055576; https://center6.umin.ac.jp/cgi-open-bin/ctr_e/ctr_view.cgi?recptno=R000063507).

### 2.2 Participants

The inclusion criteria were as follows: (1) individuals who received a sufficient explanation of the purpose and procedures of this study, had the capacity to provide informed consent, fully understood the information provided, and voluntarily agreed to participate; (2) healthy adult women aged 20–39 years at the time of obtaining informed consent; (3) individuals with a menstrual cycle length ranging from 25 to 38 days, calculated based on the previous six menstrual cycles, and a usual menstrual duration of 3–7 days; (4) individuals who experienced subjective menstruation-related symptoms at any time from 3 days before the onset of menstruation to the third day of menstruation.

The exclusion criteria were as follows: (1) individuals who were pregnant, breastfeeding, or possibly pregnant; (2) individuals who intended to become pregnant during the study period; (3) individuals with a history or current diagnosis of gynecological diseases (including secondary amenorrhea, dysmenorrhea, endometriosis, uterine fibroids, PMDD, breast cancer, cervical cancer, endometrial cancer, or ovarian cancer); (4) individuals with allergies to milk, wheat, eggs, shrimp, crab, peanuts, or walnuts, or those with lactose intolerance, because these ingredients or related allergens may be present in the test foods; (5) individuals who had participated in another clinical trial or monitoring study within 2 months prior to obtaining informed consent; (6) individuals who had no menstrual pain or whose menstrual pain was so severe that it could not be controlled with over-the-counter analgesics; (7) individuals who regularly used medications (including traditional Japanese herbal medicines and oral contraceptives) or supplements that could potentially affect the study outcomes during the 2 months before consent; (8) individuals with a history or current diagnosis of mood disorders, or those with a score of 60 or higher on the Self-Rating Depression Scale (SDS) (29); (9) individuals classified as level IV in the neuroticism domain according to the Cornell Medical Index Health Questionnaire, Japanese version (J-CMI) (30); (10) individuals with a score of 20 or higher on the Stress Checklist KM (SCL-KM) (31).

### 2.3 Study procedures

The study was conducted at Kayaba-cho Mental Health Care Clinic (Tokyo). Participants were recruited through a volunteer bank and consisted of healthy Japanese women. All procedures, including the explanation of the study, acquisition of informed consent, administration of questionnaires, and examinations, were conducted in Japanese. After receiving a full explanation of the study, participants provided electronic informed consent.

Participants visited the clinic and underwent a screening examination (SCR), which included the Menstrual Distress Questionnaire (MDQ), J-CMI, SDS, SCL-KM, physical measurements (height and weight), blood pressure measurement, blood sampling, and a medical interview conducted by a physician to assess eligibility. Blood samples were collected using the clinic’s standard procedures, and routine hematological and biochemical tests were performed (see Supplementary Table S1 for the list of test items). If the number of eligible participants exceeded the target sample size of 140, 140 participants were enrolled in order of proximity of their total MDQ scores to the median among all eligible participants.

The allocation manager generated a computer-based randomization sequence using age and pre-intervention MDQ scores as stratification factors and randomly assigned participants to one of the two groups at a 1:1 ratio (*n* = 70 per group). The allocation manager prepared an allocation list containing participant IDs and test food codes; the list was immediately sealed and stored under lock and key and was revealed only after the final data lock. Consequently, blinding was maintained for all participants and investigators except the allocation manager. The allocation manager was not involved in operations other than the allocation and confirmation that the test foods were indistinguishable.

**Figure 1:**
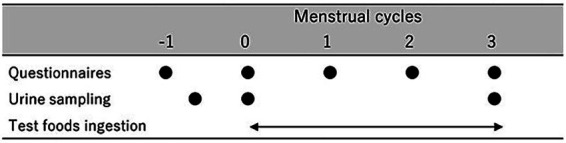
Study schedule.

Participants completed questionnaires on five occasions, from the first menstruation following the SCR (defined as menstrual cycle −1) through the fifth menstruation (defined as menstrual cycle 3) (Figure 1). The questionnaires included the MDQ, SF-36, and a visual analog scale (VAS). Participants completed the questionnaires on the first day of menstruation while recalling the preceding 3 days, and again on day 4 of menstruation while recalling the preceding 3 days. These assessments were defined as evaluations of the premenstrual phase and the menstrual phase, respectively. Assessments obtained during menstrual cycle −1 were used as baseline values, and these data were applied to stratified randomization.

Participants collected urine samples on three occasions in total: on days 8–14 of menstrual cycle −1, on day 2 of menstruation in the menstrual cycle 0, and on day 2 of menstruation in the menstrual cycle 3. Participants consumed either a powdered food containing aLA (900 mg/day) or a placebo powdered food (in which dextrin was used instead of aLA) once daily for three menstrual cycles, from day 8 of menstruation in cycle 0 to day 4 of menstruation in cycle 3. The powdered test foods were dissolved in water before consumption. The timing of daily intake was left to the participants’ discretion.

Participants used a smartphone application to keep a diary every day. In addition, participants were instructed to comply with the following prohibitions during the study period: (1) not to travel overseas or domestically for more than 1 week; (2) not to take analgesics as a prophylactic measure prior to the onset of menstrual pain (the use of analgesics for pain relief after the pain onset was permitted); (3) not to consume alcohol on the day before the SCR; and not to eat or drink anything other than water from 12 h before the SCR if it was conducted in the morning, or from 6 h before the SCR if it was conducted in the afternoon.

### 2.4 Test foods

The test foods were manufactured by API Co., Ltd. (Gifu, Japan) under the direction of the test-food manufacturing supervisor, Meiji Co., Ltd. (Hachioji, Tokyo). The aLA food was formulated to contain 900 mg of aLA per unit. The placebo food was manufactured as a powder containing dextrin. Each food was individually packaged in plain aluminum pouches, 1 day’s supply per pouch, such that neither the participants nor the investigators could distinguish between them. In addition, the allocation manager confirmed that the placebo and the aLA food could not be distinguished. Each test food was packed in cardboard boxes and sent to the allocation manager in a randomly coded state. The test-food manufacturing supervisor prepared a correspondence table linking the codes to the types of test foods and kept it sealed and stored until unblinding. The test-food manufacturing supervisor was not involved in any operations other than those related to the preparation of the test foods.

### 2.5 Questionnaires

#### 2.5.1 Menstruation-related symptoms

Menstruation-related symptoms were assessed using the MDQ. The original MDQ was developed by Moos et al. (32). The reliability and validity of the MDQ have been widely demonstrated, and it is one of the most commonly used self-report instruments for evaluating the severity of menstruation-related symptoms. In the present study, based on studies indicating low relevance among Japanese populations (33, 34), the “Arousal” and “Control” factors were excluded. Consequently, the questionnaire consisted of 35 items across six factors. The 35-item version of the MDQ has been frequently used in previous studies involving Japanese participants (25, 35, 36). Each item was rated on a scale from 0 (no symptoms) to 4 (very severe symptoms). Scores were summed for each of the six factors as well as for all items combined.

#### 2.5.2 Health-related QoL

Health-related QoL was assessed using the Japanese version of the 36-Item Short Form Health Survey, Version 2 (SF-36) (37, 38). The SF-36 is widely used internationally to measure QoL. It evaluates the following eight health concepts: physical functioning (PF), limitations on role functioning because of physical health (RP), bodily pain (BP), general health (GH), vitality (VT), social functioning (SF), limitations in role functioning because of emotional problems (RE), and mental health (MH). In this study, physical component summary (PCS), mental component summary (MCS), and role/social component summary (RCS) scores were calculated in accordance with the three-component scoring method (39).

#### 2.5.3 VAS

During the premenstrual phase, the following 12 symptoms were assessed using VAS: depressed mood, anxiety, mood swings, irritability, decreased interest in activities, difficulty concentrating, easy fatigability, overeating, inability to sleep, sleeping more than usual, feeling out of control, and physical symptoms (breast pain, fullness, and swelling). During menstruation, the following 10 symptoms were assessed using VAS: lower abdominal pain, back pain, headache, muscle discomfort, fatigue, irritability, skin irritation, depression, insomnia, and inability to concentrate. Participants recorded symptom severity on a 100-mm scale ranging from 0 mm (no symptoms) to 100 mm (severe symptoms).

The VAS for premenstrual symptoms was based on a questionnaire developed by Steiner et al. (40) in accordance with the diagnostic criteria for premenstrual dysphoric disorder defined by the American Psychiatric Association. The validity of this VAS using the same questionnaire items has been reported in a Japanese population (41). Since there was no standardized VAS questionnaire for assessing menstrual symptoms, the authors developed original items.

#### 2.5.4 Frequency of analgesic use

The frequency of analgesic use was scored for each premenstrual and menstrual phase as 0 (no use), 1 (one use), 2 (two uses), and 3 (three or more uses).

### 2.6 Measurements of urinary PGs

The major PGs produced in the endometrium are PGF2α and PGE2, and their association with menstrual pain has been reported (11–13). In addition, observational studies have reported that urinary PGF2α is associated with dysmenorrhea (42, 43). Because PGs are metabolized *in vivo* (44), their urinary metabolites were also measured.

Urine samples were collected as follows: after inserting a tampon, participants thoroughly washed away menstrual blood in the shower and then collected urine to avoid contamination by menstrual blood. Urinary levels of PGF2α, PGE2, the PGF2α metabolite, and the PGE2 metabolite were measured using enzyme-linked immunosorbent assays (ELISA) (Item Nos. 516,011, 514,010, 516,671, and 514,531, respectively). All ELISA kits were purchased from Cayman Chemical (Ann Arbor, MI) and the assays were performed according to the manufacturer’s instructions. Urinary PG levels were adjusted for urinary creatinine levels.

### 2.7 Outcomes

The primary outcome was the MDQ. The secondary outcomes were the SF-36, VAS, and urinary PGs.

### 2.8 Adverse events

In this study, an adverse event (AE) was defined as any newly occurring symptom or disease that developed between the start of test-food intake and the end of the study. AEs were reported based on participants’ daily diary entries. Classification of AE severity and its relationship to the study was assessed by the principal investigator. The severity of AEs was classified as follows: “mild,” events that were easily tolerated and did not interfere with daily activities; “moderate,” events that caused some interference with daily activities; and “severe,” events that prevented normal daily activities.

### 2.9 Statistical analysis

The sample size was calculated as follows: Based on the first-period results of a previous crossover study (25), the effect size for a parallel-group comparison was estimated to be approximately 0.3. The present study employed a repeated-measures design and aimed to comprehensively evaluate outcomes at three post-intake time points. Taking into account the design effect associated with the repeated measurements (45), the required sample size was calculated to achieve a two-sided significance level of 5% and a statistical power of 80%. Assuming a discontinuation or dropout rate of approximately 10%, the target sample size was set at 70 participants per group, for a total of 140 participants.

All efficacy analyses were conducted using the per-protocol set (PPS) in accordance with the prespecified protocol, because the primary objective of this study was to evaluate the efficacy of the test food under conditions of adequate adherence and protocol compliance. No multiplicity adjustment was applied, as analyses beyond the primary outcome were considered exploratory. The PPS excluded participants who met any of the following criteria: (1) compliance with intake of the test food was less than 80%; (2) violation of the study prohibitions; (3) the principal investigator judged that the reliability of the test results was compromised due to conspicuous inappropriate behavior; or (4) the principal investigator determined, for other reasonable reasons, that the participant was unsuitable for inclusion in the analysis. The Safety Analysis Set (SAS) included all participants who consumed the test food at least once.

Baseline characteristics of the participants were summarized using means and standard deviations (SDs), or numbers and percentages, as appropriate.

The efficacy of the test food for MDQ, SF-36, and VAS outcomes was evaluated using a mixed-effects model for repeated measures (MMRM). The dependent variable was defined as the change from baseline at each post-intake time point. Fixed effects included group, time point, and the group-by-time interaction. Baseline value, age, and body mass index (BMI) were included as covariates in the model. An unstructured covariance matrix was assumed to account for within-subject correlations arising from repeated measurements. Missing data were assumed to be missing at random, and no imputation was performed. The effect of the test food was assessed based on the group main effect and the group-by-time interaction effect. Between-group comparisons at each time point were conducted only when the group-by-time interaction effect was statistically significant. Least squares means (LSMs) and their standard errors (SEs) were estimated from the model.

Urinary PGs were summarized as means and SEs. Between-group comparisons were conducted using Student’s t-test, and within-group comparisons were performed using paired *t*-tests.

For safety evaluation, the number of participants experiencing AEs was tabulated. The incidence of AEs, overall and by severity, was compared between groups using Fisher’s exact test.

All statistical analyses were performed using two-sided tests with a significance level of 5%. Statistical analyses were conducted using Microsoft Excel for Microsoft 365 and R (version 4.3.1).

## 3 Results

### 3.1 Participant Enrollment, baseline characteristics, and study compliance

Participant recruitment for this study began on October 1, 2024, and follow-up was completed on July 10, 2025.

**Figure 2:**
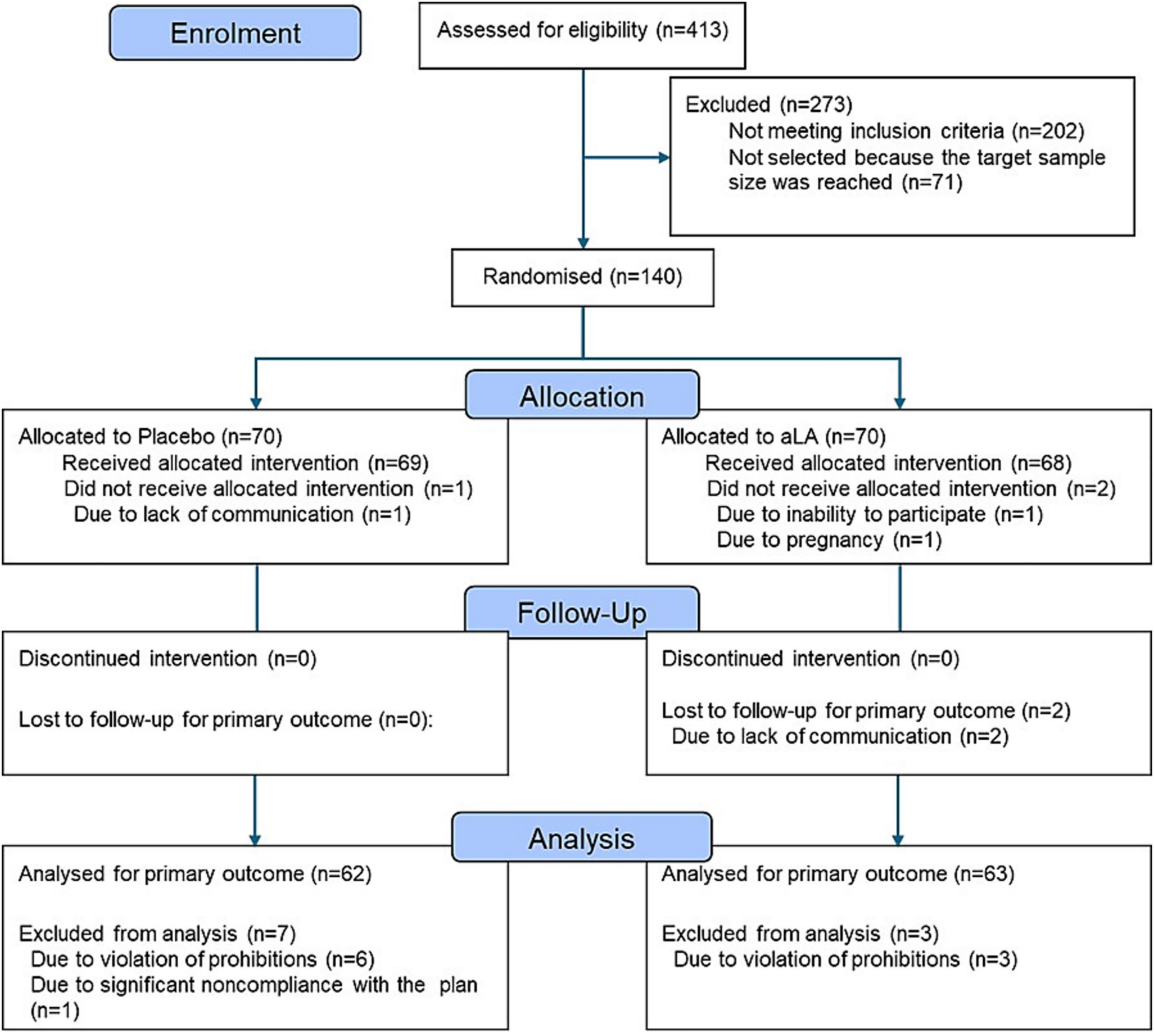
CONSORT flow diagram.

The flow of participants through the study is shown in Figure 2. A total of 413 Japanese women who provided informed consent underwent the SCR. Of these, 202 participants were excluded because they did not meet the eligibility criteria. As the number of eligible participants exceeded the target sample size, an additional 71 participants were not selected. Consequently, 140 participants were selected and randomly assigned in a 1:1 ratio to either the placebo group or the aLA group.

Before the initiation of the intervention, one participant in the placebo group and two participants in the aLA group withdrew from the study due to loss of contact, voluntary withdrawal, or pregnancy. During the intervention period, two additional participants in the aLA group discontinued the study because of loss of contact. Among those who completed the intervention, seven participants in the placebo group and three in the aLA group were excluded from the efficacy analysis due to protocol violations (overseas travel, long-term domestic travel, or markedly low compliance with the test food). As a result, 62 participants in the placebo group and 63 participants in the aLA group were included in the PPS for efficacy analysis.

Baseline characteristics of the participants are presented in Table 1 and Supplementary Table S1.

**Table 1:** Baseline characteristics of the participants (PPS).

Item	Unit	Placebo *n* = 62	aLA *n* = 63
Age	years	32.1 ± 5.3	32.3 ± 5.4
Height	cm	158.5 ± 4.6	159.7 ± 4.8
Weight	kg	53.4 ± 6.8	52.9 ± 7.1
BMI	kg/m^2^	21.2 ± 2.3	20.8 ± 2.7
Menstrual cycle	days	30.4 ± 2.5	30.1 ± 2.2
Menstrual phase	days	5.3 ± 1.2	5.5 ± 1.1
Stress score		5.7 ± 4.1	5.9 ± 4.1
MDQ total score			
Premenstrual		34.3 ± 19.9	30.3 ± 18.2
During menstruation		36.1 ± 19.7	32 ± 17.3
After menstruation		6.3 ± 7.4	7.2 ± 10.7
J-CMI			
I		38 (61.3)	43 (68.3)
II		22 (35.5)	17 (27.0)
III		2 (3.3)	3 (4.8)

Data are shown as mean ± SD, or number (percentage). aLA, alpha-lactalbumin; BMI, body mass index; MDQ, menstrual distress questionnaire; J-CMI, the Japanese edition of the Cornell Medical Index Health Questionnaire.

The mean compliance rates for the test foods were 98.4 and 98.5%, respectively, in the aLA and placebo groups.

Concomitant use of analgesics was scored based on frequency of use and compared between groups for each menstrual cycle. No significant between-group differences were observed in any menstrual cycle.

### 3.2 MDQ

Tables 2 and 3 present the results for the primary outcome, namely the total score and the scores for each factor of the MDQ. During the premenstrual phase, a statistically significant group-by-time interaction effect was observed for “Autonomic reactions” (*p* = 0.0350). However, because no significant between-group differences were observed at any individual time point, this finding was considered to have limited clinical relevance. No significant group-by-time interaction effects or group main effects were observed for the other items. During the menstrual phase, no significant group-by-time interactions or group main effects were observed for any of the items.

**Table 2:** Changes of MDQ scores during the premenstrual phase.

Factors	Group	LSM ± SE			MMRM, *p* value				
		Cycle 1	Cycle 2	Cycle 3	Group×Time interaction	Group main effect	Cycle 1	Cycle 2	Cycle 3
Total	Placebo	−2.6 ± 2.0	−5.0 ± 2.3	−4.5 ± 2.4	0.1628	0.4661	NA	NA	NA
	aLA	−6.1 ± 2.0	−4.2 ± 2.3	−7.8 ± 2.4					
Pain	Placebo	−1.3 ± 0.4	−1.8 ± 0.5	−1.9 ± 0.5	0.1078	0.6333	NA	NA	NA
	aLA	−1.3 ± 0.4	−0.7 ± 0.5	−2.2 ± 0.5					
Water retention	Placebo	−0.9 ± 0.3	−0.9 ± 0.3	−0.7 ± 0.3	0.0895	0.5306	NA	NA	NA
	aLA	−1.0 ± 0.3	−0.6 ± 0.3	−1.4 ± 0.3					
Autonomic reactions	Placebo	0.4 ± 0.2	0.1 ± 0.2	0.3 ± 0.2	**0.0350**	0.8539	0.2040	0.2266	0.7345
	aLA	0.0 ± 0.2	0.4 ± 0.2	0.1 ± 0.2					
Negative affect	Placebo	−0.8 ± 0.7	−1.1 ± 0.7	−0.9 ± 0.7	0.7968	0.5278	NA	NA	NA
	aLA	−1.1 ± 0.7	−1.4 ± 0.7	−1.9 ± 0.7					
Concentration	Placebo	0.7 ± 0.6	0.1 ± 0.6	−0.1 ± 0.6	0.3710	0.1956	NA	NA	NA
	aLA	−0.9 ± 0.6	−0.5 ± 0.6	−0.9 ± 0.7					
Behavioral change	Placebo	−0.6 ± 0.4	−1.3 ± 0.5	−1.0 ± 0.5	0.2235	0.2297	NA	NA	NA
	aLA	−1.8 ± 0.4	−1.4 ± 0.5	−1.7 ± 0.5					

Values are presented as LSM±SE. MMRM analyses were conducted. Group comparisons at each time point conducted when the group-by-time interaction effect was significant. aLA, alpha-lactalbumin; MDQ, menstrual distress questionnaire; MMRM, mixed-effects models with repeated measures; NA, not assessed.

**Table 3:** Changes of MDQ scores during the menstrual phase.

Factors	Group	LSM ± SE			MMRM, *p* value				
		Cycle 1	Cycle 2	Cycle 3	Group×Time interaction	Group main effect	Cycle 1	Cycle 2	Cycle 3
Total	Placebo	−0.7 ± 1.9	−1.8 ± 2.1	−3.4 ± 2.0	0.8976	0.9058	NA	NA	NA
	aLA	−1.5 ± 1.9	−2.0 ± 2.0	−3.2 ± 2.0					
Pain	Placebo	−0.3 ± 0.5	−0.7 ± 0.5	−1.2 ± 0.5	0.6853	0.7727	NA	NA	NA
	aLA	−0.3 ± 0.5	−1.2 ± 0.4	−1.2 ± 0.5					
Water retention	Placebo	−0.2 ± 0.3	−0.1 ± 0.3	−0.5 ± 0.3	0.7316	0.7705	NA	NA	NA
	aLA	−0.4 ± 0.3	−0.3 ± 0.3	−0.4 ± 0.3					
Autonomic reactions	Placebo	0.2 ± 0.2	−0.1 ± 0.2	0.0 ± 0.2	0.2532	0.8174	NA	NA	NA
	aLA	0.0 ± 0.2	0.2 ± 0.2	0.2 ± 0.2					
Negative affect	Placebo	0.1 ± 0.5	0.2 ± 0.6	0.0 ± 0.6	0.7698	0.7196	NA	NA	NA
	aLA	0.1 ± 0.5	0.0 ± 0.6	−0.5 ± 0.6					
Concentration	Placebo	0.5 ± 0.6	−0.3 ± 0.5	−0.4 ± 0.5	0.1671	0.9558	NA	NA	NA
	aLA	−0.2 ± 0.6	0.2 ± 0.5	−0.1 ± 0.5					
Behavioral change	Placebo	−0.9 ± 0.4	−0.9 ± 0.4	−1.4 ± 0.4	0.8110	0.8906	NA	NA	NA
	aLA	−0.8 ± 0.4	−1.0 ± 0.4	−1.2 ± 0.4					

Values are presented as LSM±SE. MMRM analyses were conducted. Group comparisons at each time point were performed when the group-by-time interaction effect was significant. aLA, alpha-lactalbumin; MDQ, menstrual distress questionnaire; MMRM, mixed-effects models with repeated measures; NA, not assessed.

As an exploratory analysis, the results for the MDQ subscale items are presented in Supplementary Tables S2 and S3. During the premenstrual phase, significant group main effects were observed for “Weight gain” and “Lowered motor coordination (difficulty moving the body as intended)” (*p* = 0.0202 and *p* = 0.0250, respectively), while no significant group-by-time interaction effects were observed for either item. For “General aches and pains,” a significant group-by-time interaction effect was observed (*p* = 0.0444); however, because no significant between-group differences were observed at any individual time point, this finding was considered to have limited clinical relevance. No significant group-by-time interaction effects or group main effects were observed for the other items.

During the menstrual phase, significant group-by-time interaction effects were observed for “Nausea, vomiting” and “Accidents (making a small mistake)” (*p* = 0.0230 and *p* = 0.0246, respectively). However, as no significant between-group differences were observed at any time point for either item, these findings were considered to have limited clinical relevance. No significant group-by-time interaction effects or group main effects were observed for the other items.

### 3.3 Health-related QoL

**Figure 3:**
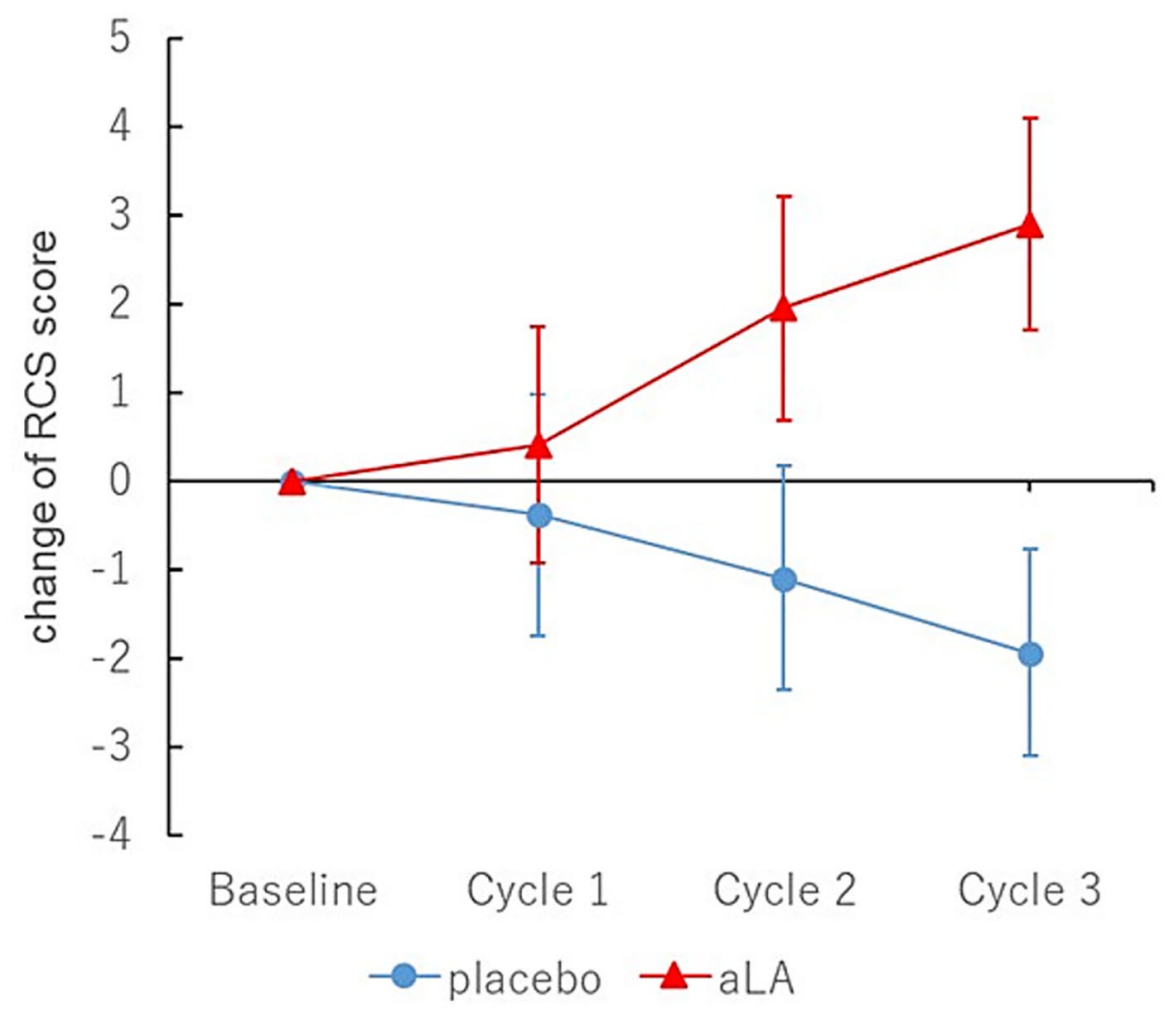
Changes of RCS in SF-36 during the premenstrual phase. Values are presented as LSM ± SE. MMRM analyses were conducted. MMRM analysis showed a significant group main effect (*p* = 0.0491). aLA, alpha-lactalbumin; RCS, role/social component summary; MMRM, mixed-effects models with repeated measures.

Tables 4 and 5 present the results of the SF-36. During the premenstrual phase, RCS showed a significant group main effect (*p* = 0.0491, see also Figure 3), whereas no significant group-by-time interaction effect was observed. The adjusted mean difference averaged over time was 2.9 points, and the corresponding standardized effect size was small (Hedges’ g = 0.18). In contrast, PCS showed a statistically significant group-by-time interaction effect (*p* = 0.0011). In the between-group comparisons at each time point, the aLA group showed significantly higher values than the placebo group after one cycle of intake (*p* = 0.0210). However, because no significant between-group differences were observed after two or three cycles of intake, the clinical relevance of long-term intake was considered to be limited.

**Table 4:** Changes of SF-36 scores during the premenstrual phase.

Factors	Group	LSM ± SE			MMRM, *P* value				
		Cycle 1	Cycle 2	Cycle 3	Group×Time interaction	Group main effect	Cycle 1	Cycle 2	Cycle 3
PCS	Placebo	−0.9 ± 0.9	1.5 ± 0.8	2.6 ± 0.9	**0.0011**	0.9395	**0.0210**	0.5816	0.0863
	aLA	2.2 ± 0.9	0.8 ± 0.8	0.4 ± 0.9					
MCS	Placebo	1.7 ± 0.9	1.0 ± 1.0	0.9 ± 1.1	0.2673	0.7781	NA	NA	NA
	aLA	0.2 ± 0.9	0.3 ± 1.0	2.0 ± 1.1					
RCS	Placebo	−0.4 ± 1.4	−1.1 ± 1.3	−1.9 ± 1.2	0.1140	**0.0491**	NA	NA	NA
	aLA	0.4 ± 1.3	2.0 ± 1.3	2.9 ± 1.2					
PF	Placebo	0.1 ± 0.7	1.7 ± 0.7	1.9 ± 0.7	0.4596	0.4578	NA	NA	NA
	aLA	1.4 ± 0.7	2.2 ± 0.7	1.9 ± 0.7					
RP	Placebo	−1.5 ± 1.1	−0.3 ± 1.1	0.1 ± 1.0	0.4717	0.0945	NA	NA	NA
	aLA	1.6 ± 1.1	0.8 ± 1.1	2.2 ± 1.0					
BP	Placebo	−0.1 ± 1.2	1.5 ± 1.1	2.7 ± 1.2	0.1601	0.2933	NA	NA	NA
	aLA	3.2 ± 1.2	1.8 ± 1.1	3.3 ± 1.2					
GH	Placebo	0.6 ± 0.8	0.4 ± 0.9	1.0 ± 0.9	0.5240	0.5225	NA	NA	NA
	aLA	0.6 ± 0.7	−0.4 ± 0.9	−0.3 ± 0.9					
VT	Placebo	1.3 ± 1.1	1.6 ± 1.1	1.3 ± 1.1	0.0965	0.9630	NA	NA	NA
	aLA	−0.3 ± 1.0	1.0 ± 1.1	3.4 ± 1.1					
SF	Placebo	0.6 ± 1.3	0.8 ± 1.1	1.0 ± 1.1	0.2143	0.1756	NA	NA	NA
	aLA	0.4 ± 1.2	3.1 ± 1.1	4.1 ± 1.1					
RE	Placebo	0.0 ± 1.2	−1.1 ± 1.2	−1.8 ± 1.2	0.3993	0.0620	NA	NA	NA
	aLA	1.2 ± 1.2	1.5 ± 1.2	2.1 ± 1.2					
MH	Placebo	1.2 ± 1.0	0.0 ± 1.2	−0.6 ± 1.1	0.2459	0.3192	NA	NA	NA
	aLA	1.1 ± 1.0	0.7 ± 1.2	2.3 ± 1.2					

Values are presented as LSM±SE. MMRM analyses were conducted. Group comparisons at each time point were performed when the group-by-time interaction effect was significant. aLA, alpha-lactalbumin; SF-36, the Japanese version of the 36-Item Short Form Health Survey, Version 2; PCS, physical component summary; MCS, mental component summary; RCS, role/social component summary; PF, physical functioning; RP, role physical; BP, bodily pain; GH, general health; VT, vitality; SF, social functioning; RE, role emotional; MH, mental health; MMRM, mixed-effects models with repeated measures; NA, not assessed.

**Table 5:** Changes of SF-36 scores during the menstrual phase.

Factors	Group	LSM ± SE			MMRM, *P* value				
		Cycle 1	Cycle 2	Cycle 3	Group×Time interaction	Group main effect	Cycle 1	Cycle 2	Cycle 3
PCS	Placebo	0.4 ± 1.0	0.3 ± 0.8	0.0 ± 0.9	0.7266	0.9145	NA	NA	NA
	aLA	0.6 ± 1.0	−0.1 ± 0.8	0.6 ± 0.9					
MCS	Placebo	0.3 ± 0.9	−1.1 ± 1.0	0.2 ± 1.1	0.5644	0.8899	NA	NA	NA
	aLA	−0.4 ± 0.9	−0.3 ± 1.0	0.5 ± 1.1					
RCS	Placebo	0.6 ± 1.2	4.0 ± 1.1	1.7 ± 1.2	0.3586	0.5462	NA	NA	NA
	aLA	2.7 ± 1.1	3.7 ± 1.1	2.3 ± 1.2					
PF	Placebo	0.1 ± 1.0	1.7 ± 0.7	0.4 ± 1.0	0.7765	0.6619	NA	NA	NA
	aLA	0.6 ± 0.9	1.6 ± 0.7	1.2 ± 1.0					
RP	Placebo	1.1 ± 1.1	2.1 ± 1.1	0.5 ± 1.1	0.4603	0.5169	NA	NA	NA
	aLA	2.4 ± 1.1	1.8 ± 1.1	2.0 ± 1.1					
BP	Placebo	1.4 ± 1.3	0.8 ± 1.1	2.3 ± 1.2	0.7968	0.2945	NA	NA	NA
	aLA	2.9 ± 1.3	2.8 ± 1.1	3.3 ± 1.2					
GH	Placebo	−0.4 ± 0.9	−1.7 ± 0.9	−1.5 ± 0.8	0.8539	0.8061	NA	NA	NA
	aLA	−0.9 ± 0.9	−2.1 ± 0.9	−1.4 ± 0.8					
VT	Placebo	0.3 ± 1.0	1.0 ± 1.0	0.8 ± 1.1	0.7289	0.5445	NA	NA	NA
	aLA	1.0 ± 1.0	1.2 ± 1.0	2.2 ± 1.2					
SF	Placebo	−0.3 ± 1.1	1.0 ± 1.0	1.6 ± 1.2	0.4603	0.3183	NA	NA	NA
	aLA	1.5 ± 1.1	2.8 ± 1.0	1.6 ± 1.2					
RE	Placebo	0.3 ± 1.1	3.6 ± 1.0	0.8 ± 1.0	0.0959	0.4024	NA	NA	NA
	aLA	2.6 ± 1.0	2.9 ± 1.0	2.2 ± 1.1					
MH	Placebo	1.1 ± 0.9	0.6 ± 1.1	0.4 ± 1.1	0.6258	0.8314	NA	NA	NA
	aLA	0.4 ± 0.9	1.2 ± 1.0	1.2 ± 1.1					

Values are presented as LSM±SE. MMRM analyses were conducted. Group comparisons at each time point were performed when the group-by-time interaction effect was significant. aLA, alpha-lactalbumin; SF-36, the Japanese version of the 36-Item Short Form Health Survey, Version 2; PCS, physical component summary; MCS, mental component summary; RCS, role/social component summary; PF, physical functioning; RP, role physical; BP, bodily pain; GH, general health; VT, vitality; SF, social functioning; RE, role emotional; MH, mental health; MMRM, mixed-effects models with repeated measures; NA, not assessed.

During the menstrual phase, no significant group-by-time interaction effects or group main effects were observed for any of the items.

### 3.4 VAS

The results of the VAS are presented in Supplementary Tables S4 and S5. In both the premenstrual and menstrual phases, no significant group-by-time interaction effects or group main effects were observed for any of the items.

### 3.5 PGs

The results of the urinary PGs are presented in Supplementary Table S6. No significant between-group differences were observed for any of the items. PGF2α metabolites showed significantly higher levels during the pre-intake menstrual phase than during the pre-intake non-menstrual phase in both groups (*p* < 0.05 for each group). PGE2 metabolites showed significantly higher levels during the post-intake menstrual phase than during the pre-intake menstrual phase in both groups (*p* < 0.05 for each group).

### 3.6 AEs

The SAS comprised 68 participants in the aLA group and 69 participants in the placebo group, corresponding to participants classified as “Received allocated intervention” in Figure 2. The number and incidence of AEs in each group are shown in Table 6, and the details of the AEs are presented in Supplementary Table S7. AEs were observed in 32 participants in the aLA group and 29 participants in the placebo group. According to Fisher’s exact test, no significant between-group difference was observed in overall AE incidence (*p* = 0.6078). In addition, no significant between-group differences were observed when AEs were classified by severity (mild or moderate; *p* = 0.3907 and *p* = 0.7183, respectively). No serious AEs were reported. All observed AEs were considered to be transient or incidental, and the principal investigator judged that there was no causal relationship between the study and the AEs.

**Table 6:** AEs.

Item	Total		Mild		Moderate		Severe	
	aLA	Placebo	aLA	Placebo	aLA	Placebo	aLA	Placebo
Adverse events	32 (47.1)	29 (42.0)	32 (47.1)	27 (39.1)	4 (5.9)	3 (4.3)	0 (0)	0 (0)
*p*-value	0.6078		0.3907		0.7183		–	

The number and percentage of participants with AEs is presented. AE incidence was compared between the aLA and placebo groups using Fisher’s exact test. No serious AEs or study-related AEs were observed. aLA, alpha-lactalbumin.

## 4 Discussion

The present study aimed to evaluate the effects of long-term aLA intake on menstruation-related symptoms in healthy Japanese women. In the primary outcome, the MDQ, no significant between-group differences were observed in either the total score or the six factor scores during the premenstrual phase or menstrual phase. On the other hand, exploratory analyses suggested that aLA may lead to significant improvements in the premenstrual symptoms of “Weight gain” and “Lowered motor coordination.” Furthermore, in the secondary outcomes, aLA significantly improved the RCS score of the SF-36 during the premenstrual phase. However, the clinical significance of this finding warrants caution. The mean difference in the RCS score was 2.9 points, corresponding to a small effect size. To our knowledge, the minimal clinically important difference for the RCS has not been established. Although these secondary and exploratory findings should be interpreted with caution, they raise the hypothesis that long-term aLA intake could improve role-related and social aspects of quality of life, possibly by alleviating specific premenstrual symptoms. This hypothesis requires testing in future studies.

The present study investigated the long-term intake of aLA and observed differences in efficacy compared with previous studies examining short-term intake. One previous study reported that aLA intake for one menstrual cycle alleviated menstrual pain, although no significant between-group differences were observed compared with the placebo group (24). Another study reported that aLA intake for one menstrual cycle significantly reduced physical symptom scores during menstruation (25). Based on these previous findings, in the present study we expected aLA to be effective particularly for the pain factor of the MDQ during menstruation; however, no significant between-group differences were observed.

One possible explanation for the discrepancy with previous studies is a stronger placebo effect over time. In the present study, it is conceivable that long-term intake led to a gradual increase in placebo responses with each menstrual cycle, attenuating the between-group differences. Previous studies have also suggested a strong placebo effect on menstruation-related symptoms. It has been reported that approximately 20% of women with PMS experienced a sustained improvement in symptoms of 50% or more with placebo administration alone (46), indicating that placebo effects can be pronounced in premenstrual symptoms and may strengthen over time. Placebo effects have also been observed in intervention studies of menstrual pain (24). In addition, although not specific to menstrual pain, studies on analgesic effects have reported that placebo effects can be large and sustained over time (47). In the present study, a significant improvement with aLA was observed only after one cycle of intake in the PCS, the physical summary score of the SF-36, during the premenstrual phase; however, the difference disappeared after the second cycle. This finding also could be consistent with the possibility that the placebo effect increased over time.

Environmental factors affecting menstruation-related symptoms may also have increased variability, making the effect of the test food more difficult to detect. Menstruation-related symptoms have been reported to be influenced not only by lifestyle factors such as diet (48), bathing habits (48), and sleep quality (49), but also by psychological stress, including high job demands and low coworker support (50). The present study was conducted across April, a period in Japan during which personnel transfers and changes in work responsibilities frequently occur. Such timing may have affected menstruation-related symptoms through changes in lifestyle and work-related stress, thereby increasing variability.

As a potential mechanism underlying the finding in premenstrual role-related and social QoL associated with aLA, an effect mediated by Trp, a precursor of serotonin, may be considered. aLA has an amino acid composition rich in Trp. In a previous study, 20 g/day of aLA intake increased the Trp ratio in plasma, which was associated with improved mood in stress-sensitive participants (16). In addition, a previous study reported that a daily intake of 6 g of Trp was effective in women with PMDD (51). On the other hand, the aLA dose used in this study (900 mg/day) is equivalent to approximately 43 mg/day of Trp (assuming a Trp content of 48 mg/g in aLA (52)), which is extremely low compared with the 6 g/day Trp dose used in the PMDD study (51). Moreover, the aLA dose in the present study was substantially lower than that used in the previous study, in which participants consumed 20 g/day of aLA (16). Therefore, the effects of aLA observed in this study cannot be explained solely by Trp, suggesting that other mechanisms are likely involved.

Another potential mechanism underlying the effects of aLA is its anti-inflammatory activity; however, direct evidence to support this hypothesis was not obtained in the present study. Observational studies in women with PMS have shown that elevations in multiple cytokines are associated with PMS severity (53), suggesting the involvement of inflammation in PMS. In addition, an association between high-sensitivity C-reactive protein, an inflammatory marker, and weight gain/bloating has been reported in women with PMS (54). In animal studies, aLA administration has been reported to exert anti-inflammatory and analgesic effects (19–21), thus we hypothesized that aLA might alleviate PMS symptoms in our study. However, this study did not assess inflammatory markers such as plasma cytokines or CRP, because blood was not collected to minimize participant burden. Moreover, no significant between-group differences were observed in urinary PGs, which are considered objective biomarkers. This outcome may suggest that the anti-inflammatory effect of aLA was too subtle to be detected under our study conditions, or that urinary measurements had limited sensitivity. Future research should elucidate the mechanism of action using more sensitive evaluation methods, such as direct measurement of plasma cytokines and CRP as well as analysis of PGs in menstrual fluid to more directly reflect endometrial conditions than urinary measurements (11, 13, 55).

On the other hand, it is noteworthy that the urinary concentration of the PGF2α metabolite was approximately one order of magnitude higher than that of other PGs and showed a significant increase during menstruation. Although further investigation is required, measurement of urinary PGF2α metabolites may be useful as an objective indicator of menstrual symptoms with low participant burden in large-scale observational studies and pharmaceutical intervention trials.

This study has several limitations. First, efficacy analyses were conducted using the PPS without an Intention-to-Treat (ITT) analysis. Although this prespecified approach was useful for evaluating efficacy under adequate adherence and protocol compliance, the absence of an ITT analysis may limit the robustness of the findings because the benefits of randomization may not be fully preserved after post-randomization exclusions. Second, the generalizability of our findings is constrained by the narrow study population. Because this study was designed to evaluate the intervention in a healthy population, we excluded women with clinically relevant gynecological conditions (e.g., endometriosis, severe dysmenorrhea, PMDD) and those using hormonal therapies. Consequently, our findings may not apply to women with more severe menstruation-related symptoms—arguably the population most in need of such interventions. This restriction to Japanese women aged 20 to 39 also leaves it unclear whether similar effects would be observed in other age groups or in women of different ethnic or racial backgrounds. Third, a large number of secondary and exploratory outcomes were evaluated without adjustments for multiple comparisons. As stated in the Methods section, this approach was taken because analyses beyond the primary outcome were prespecified as exploratory. Consequently, the statistically significant findings for specific outcomes, such as “Weight gain,” “Lowered motor coordination,” and RCS scores, should be interpreted with caution because they may include false positives due to chance. These results should be considered hypothesis-generating rather than confirmatory. Future studies are required to validate these preliminary findings. Fourth, dietary assessment was not conducted. As aLA is naturally consumed through dairy products, variations in participants’ dietary habits could be a potential confounding factor. While randomization aimed to minimize the impact of such variables by distributing them evenly across groups, we cannot entirely rule out the influence of background dietary aLA intake on study outcomes without dietary records. Despite these limitations, this study is meaningful in that it is the first to suggest not only the safety of long-term aLA intake but also its potential benefits in role and social QoL in healthy women during the premenstrual phase. Further evidence is expected to accumulate, supporting the establishment of aLA as a useful option for managing menstruation-related symptoms.

## 5 Conclusion

In this trial, long-term aLA supplementation did not improve overall menstruation-related symptoms as assessed by the primary outcome. However, secondary outcomes indicated potential benefits in role-related and social aspects of premenstrual QoL. These findings are exploratory, requiring further studies for confirmation.

## Data Availability

The datasets supporting the conclusions of this article will be made available by the corresponding author upon reasonable request.

## Glossary

AE: adverse event
aLA: *α*-lactalbumin
BMI: body mass index
BP: bodily pain
COX-2: cyclooxygenase-2
ELISA: enzyme linked immunosorbent assay
GABA: *γ*-aminobutyric acid
GH: general health
ITT: Intention-to-Treat
J-CMI: the Cornell Medical Index Health Questionnaire Japanese version
LSM: least squares mean
MCS: mental component summary
MDQ: Menstrual Distress Questionnaire
MH: mental health
MMRM: mixed-effects model for repeated measures
NA: not assessed
PCS: physical component summary
PF: physical functioning
PGE2: prostaglandin E2
PGF2α: prostaglandin F2α
PGs: prostaglandins
PMDD: premenstrual dysphoric disorder
PMS: premenstrual syndrome
PPS: per-protocol set
QoL: quality of life
RCS: role/social component summary
RE: limitations on role functioning because of emotional problems
RP: limitations on role functioning because of physical health
SAS: safety analysis set
SCL-KM: the Stress Checklist KM
SCR: screening examination
SD: standard deviation
SDS: Self-Rating Depression Scale
SE: standard error
SF: social functioning
SF-36: 36-Item Short Form Health Survey Version 2
SSRI: selective serotonin reuptake inhibitor
Trp: tryptophan
VAS: visual analog scale
VT: vitality.
