# Supplementary material for: Effect of long-term intake of α-lactalbumin on menstruation-related symptoms and quality of life in healthy Japanese women: a randomized, double-blind, placebo-controlled study
Source: Front Nutr. 2026 Jul 24;13:1892605. doi: 10.3389/fnut.2026.1892605 (PMC13447212; doi:10.3389/fnut.2026.1892605)
Supplement: Supplementary file 1 [file Table_1.DOCX]

Supplementary Material

**Table S1.** Baseline characteristics of the participants (Details).

| **Item** | **Unit** | **Placebo n=62** | **aLA n=63** |
| --- | --- | --- | --- |
| Systolic blood pressure | mmHg | 112.8 ± 11.1 | 110.3 ± 12.3 |
| Diastolic blood pressure | mmHg | 66.9 ± 8.7 | 67.1 ± 8.6 |
| Pulse | bpm | 75.4 ± 10.9 | 75.4 ± 9.2 |
| White blood cell | /µL | 5911 ± 1430 | 5868 ± 1585 |
| Red blood cell | ×10⁴/µL | 440.9 ± 32.0 | 436.5 ± 31.8 |
| Hemoglobin | g/dL | 13.1 ± 0.7 | 13.1 ± 0.7 |
| Hematocrit | % | 41.2 ± 2.2 | 41.3 ± 1.9 |
| Platelet count | ×10⁴/µL | 27.2 ± 4.9 | 27.3 ± 5.2 |
| Total protein | g/dL | 7.3 ± 0.4 | 7.3 ± 0.4 |
| ALB | g/dL | 4.4 ± 0.2 | 4.4 ± 0.2 |
| AST(GOT) | U/L | 18.7 ± 4.7 | 18.5 ± 7.6 |
| ALT(GPT) | U/L | 15.1 ± 9.3 | 13.4 ± 11.6 |
| γ-GT | U/L | 16.5 ± 10.6 | 16.1 ± 8.5 |
| LD | U/L | 158.8 ± 21.8 | 153.6 ± 20.5 |
| Total bilirubin | mg/dL | 0.7 ± 0.2 | 0.8 ± 0.3 |
| ALP | U/L | 57.2 ± 19.2 | 56.8 ± 11.4 |
| Urea nitrogen | mg/dL | 11.6 ± 3.9 | 11.6 ± 3.0 |
| Creatinine | mg/dL | 0.6 ± 0.1 | 0.6 ± 0.1 |
| Uric acid | mg/dL | 4.2 ± 0.9 | 4.2 ± 0.9 |
| Sodium | mEq/L | 139.5 ± 1.3 | 139.9 ± 1.2 |
| Chloride | mEq/L | 104.1 ± 2.2 | 104.1 ± 1.8 |
| Potassium | mEq/L | 4.2 ± 0.3 | 4.2 ± 0.4 |
| Calcium | mg/dL | 9.3 ± 0.3 | 9.4 ± 0.3 |
| TG (triglycerides) | mg/dL | 68.5 ± 44.4 | 62.3 ± 30.0 |
| Total cholesterol | mg/dL | 187.2 ± 24.3 | 195.9 ± 31.8 |
| HDL cholesterol | mg/dL | 71.4 ± 16.6 | 75.9 ± 16.8 |
| LDL cholesterol | mg/dL | 101.2 ± 22.0 | 106.3 ± 26.4 |
| Glucose | mg/dL | 82.2 ± 7.1 | 83.1 ± 5.8 |
| HbA1c | % | 5.2 ± 0.2 | 5.2 ± 0.2 |
| Insulin | µIU/mL | 4.6 ± 2.9 | 4.6 ± 2.5 |

Data are shown as mean ± SD or as number (percentage).

**Table S2.** Changes of MDQ subscale scores during the premenstrual phase.

|  |  | **LSM ± SE** | | | **MMRM, *P* value** | | | | |
| --- | --- | --- | --- | --- | --- | --- | --- | --- | --- |
| **Item** | **Group** | **Cycle 1** | **Cycle 2** | **Cycle 3** | **Group×Time interaction** | **Group main effect** | **Cycle 1** | **Cycle 2** | **Cycle 3** |
| Weight gain | Placebo | 0.1 ± 0.1 | 0.0± 0.1 | -0.1 ± 0.1 | 0.1652 | **0.0202** | NA | NA | NA |
|  | aLA | -0.3 ± 0.1 | -0.1 ± 0.1 | -0.3 ± 0.1 |  |  |  |  |  |
| Insomnia | Placebo | 0.2 ± 0.1 | 0.2 ± 0.1 | 0.1 ± 0.1 | 0.6900 | 0.8094 | NA | NA | NA |
|  | aLA | 0.2 ± 0.1 | 0.2 ± 0.1 | 0.2 ± 0.1 |  |  |  |  |  |
| Crying | Placebo | -0.2 ± 0.1 | -0.2 ± 0.1 | -0.2 ± 0.1 | 0.4859 | 0.3807 | NA | NA | NA |
|  | aLA | 0.0 ± 0.1 | 0.0 ± 0.1 | -0.2 ± 0.1 |  |  |  |  |  |
| Reduced school or  work performance | Placebo | 0.1 ± 0.1 | -0.2 ± 0.1 | 0.0 ± 0.1 | 0.0511 | 0.1217 | NA | NA | NA |
|  | aLA | -0.3 ± 0.1 | -0.2 ± 0.1 | -0.4 ± 0.1 |  |  |  |  |  |
| Muscle stiffness | Placebo | -0.1 ± 0.1 | -0.1 ± 0.1 | -0.3 ± 0.1 | 0.7979 | 0.6856 | NA | NA | NA |
|  | aLA | -0.1 ± 0.1 | -0.2 ± 0.1 | -0.4 ± 0.1 |  |  |  |  |  |
| Forgetfulness | Placebo | 0.2 ± 0.1 | 0.2 ± 0.1 | 0.1 ± 0.1 | 0.7678 | 0.1577 | NA | NA | NA |
|  | aLA | 0.0 ± 0.1 | 0.0 ± 0.1 | 0.0 ± 0.1 |  |  |  |  |  |
| Confusion | Placebo | 0.2 ± 0.1 | 0.1 ± 0.1 | 0.0 ± 0.1 | 0.5147 | 0.1083 | NA | NA | NA |
|  | aLA | -0.2 ± 0.1 | -0.1 ± 0.1 | -0.2 ± 0.1 |  |  |  |  |  |
| Take naps;  stay in bed | Placebo | -0.2 ± 0.1 | -0.4 ± 0.1 | -0.6 ± 0.1 | 0.2890 | 0.7968 | NA | NA | NA |
|  | aLA | -0.4 ± 0.1 | -0.4 ± 0.1 | -0.4 ± 0.1 |  |  |  |  |  |
| Headache | Placebo | -0.3 ± 0.1 | -0.2 ± 0.1 | -0.3 ± 0.1 | 0.2160 | 0.0541 | NA | NA | NA |
|  | aLA | -0.1 ± 0.1 | 0.2 ± 0.1 | -0.2 ± 0.1 |  |  |  |  |  |
| Skin disorders | Placebo | -0.2 ± 0.1 | -0.1 ± 0.1 | -0.1 ± 0.1 | 0.0687 | 0.3025 | NA | NA | NA |
|  | aLA | -0.2 ± 0.1 | -0.1 ± 0.1 | -0.5 ± 0.1 |  |  |  |  |  |
| Loneliness | Placebo | -0.1 ± 0.1 | -0.1 ± 0.1 | -0.3 ± 0.1 | 0.6501 | 0.8634 | NA | NA | NA |
|  | aLA | 0.0 ± 0.1 | -0.2 ± 0.1 | -0.2 ± 0.1 |  |  |  |  |  |
| Stay at home | Placebo | -0.2 ± 0.1 | -0.3 ± 0.1 | -0.3 ± 0.1 | 0.7826 | 0.3202 | NA | NA | NA |
|  | aLA | -0.3 ± 0.1 | -0.4 ± 0.1 | -0.5 ± 0.1 |  |  |  |  |  |
| Lower abdominal  pain | Placebo | -0.2 ± 0.1 | -0.3 ± 0.1 | -0.3 ± 0.1 | 0.6298 | 0.9257 | NA | NA | NA |
|  | aLA | -0.2 ± 0.1 | -0.2 ± 0.1 | -0.4 ± 0.1 |  |  |  |  |  |
| Dizziness, faintness | Placebo | 0.2 ± 0.1 | 0.1 ± 0.1 | 0.1 ± 0.1 | 0.1741 | 0.8253 | NA | NA | NA |
|  | aLA | 0.0 ± 0.1 | 0.2 ± 0.1 | 0.1 ± 0.1 |  |  |  |  |  |
| Avoid social activities | Placebo | 0.0 ± 0.1 | -0.1 ± 0.1 | 0.1 ± 0.1 | 0.2380 | 0.2379 | NA | NA | NA |
|  | aLA | -0.2 ± 0.1 | 0.0 ± 0.1 | -0.2 ± 0.1 |  |  |  |  |  |
| Anxiety | Placebo | -0.1 ± 0.1 | 0.0 ± 0.1 | 0.0 ± 0.1 | 0.5236 | 0.6005 | NA | NA | NA |
|  | aLA | -0.1 ± 0.1 | -0.1 ± 0.1 | -0.2 ± 0.1 |  |  |  |  |  |
| Backache | Placebo | -0.3 ± 0.1 | -0.4 ± 0.1 | -0.4 ± 0.1 | 0.4754 | 0.9640 | NA | NA | NA |
|  | aLA | -0.4 ± 0.1 | -0.3 ± 0.1 | -0.5 ± 0.1 |  |  |  |  |  |
| Cold sweats | Placebo | 0.0 ± 0.1 | 0.0 ± 0.0 | 0.1 ± 0.1 | 0.1574 | 0.6195 | NA | NA | NA |
|  | aLA | 0.0 ± 0.1 | 0.1 ± 0.0 | 0.0 ± 0.1 |  |  |  |  |  |
| Decreased judgment | Placebo | 0.0 ± 0.1 | -0.1 ± 0.1 | -0.1 ± 0.1 | 0.6287 | 0.3638 | NA | NA | NA |
|  | aLA | -0.2 ± 0.1 | -0.1 ± 0.1 | -0.2 ± 0.1 |  |  |  |  |  |
| Fatigue | Placebo | -0.3 ± 0.1 | -0.5 ± 0.1 | -0.4 ± 0.1 | 0.1476 | 0.8534 | NA | NA | NA |
|  | aLA | -0.3 ± 0.1 | -0.3 ± 0.1 | -0.5 ± 0.1 |  |  |  |  |  |
| Nausea, vomiting | Placebo | 0.1 ± 0.1 | 0.1 ± 0.1 | 0.0 ± 0.1 | 0.2837 | 0.7452 | NA | NA | NA |
|  | aLA | 0.0 ± 0.1 | 0.1 ± 0.1 | 0.0 ± 0.1 |  |  |  |  |  |
| Restlessness | Placebo | 0.1 ± 0.1 | 0.1 ± 0.1 | 0.1 ± 0.1 | 0.6219 | 0.1628 | NA | NA | NA |
|  | aLA | 0.0 ± 0.1 | 0.0 ± 0.1 | -0.1 ± 0.1 |  |  |  |  |  |
| Hot flashes | Placebo | 0.2 ± 0.1 | 0.0 ± 0.1 | 0.0 ± 0.1 | 0.2827 | 0.5944 | NA | NA | NA |
|  | aLA | 0.0 ± 0.1 | 0.0 ± 0.1 | 0.0 ± 0.1 |  |  |  |  |  |
| Difficulty  concentrating | Placebo | 0.1 ± 0.1 | -0.1 ± 0.1 | 0.0 ± 0.1 | 0.5224 | 0.2575 | NA | NA | NA |
|  | aLA | -0.2 ± 0.1 | -0.1 ± 0.1 | -0.1 ± 0.1 |  |  |  |  |  |
| Painful breasts | Placebo | -0.4 ± 0.1 | -0.4 ± 0.1 | -0.3 ± 0.1 | 0.2495 | 0.4504 | NA | NA | NA |
|  | aLA | -0.3 ± 0.1 | -0.2 ± 0.1 | -0.4 ± 0.1 |  |  |  |  |  |
| Distractibility | Placebo | 0.0 ± 0.1 | -0.1 ± 0.1 | 0.0 ± 0.1 | 0.6320 | 0.5862 | NA | NA | NA |
|  | aLA | -0.1 ± 0.1 | -0.1 ± 0.1 | -0.1 ± 0.1 |  |  |  |  |  |
| Swelling | Placebo | -0.4 ± 0.1 | -0.3 ± 0.1 | -0.3 ± 0.1 | 0.5874 | 0.7092 | NA | NA | NA |
|  | aLA | -0.3 ± 0.1 | -0.2 ± 0.1 | -0.3 ± 0.1 |  |  |  |  |  |
| Accidents | Placebo | 0.1 ± 0.1 | 0.1 ± 0.1 | 0.1 ± 0.1 | 0.6133 | 0.2574 | NA | NA | NA |
|  | aLA | -0.1 ± 0.1 | 0.1 ± 0.1 | -0.1 ± 0.1 |  |  |  |  |  |
| Irritability | Placebo | -0.2 ± 0.1 | -0.4 ± 0.1 | -0.3 ± 0.1 | 0.9918 | 0.5921 | NA | NA | NA |
|  | aLA | -0.2 ± 0.1 | -0.4 ± 0.1 | -0.3 ± 0.1 |  |  |  |  |  |
| General aches  and pains | Placebo | -0.1 ± 0.1 | -0.3 ± 0.1 | -0.3 ± 0.1 | **0.0444** | 0.9983 | 0.2047 | 0.1914 | 0.9565 |
|  | aLA | -0.3 ± 0.1 | -0.1 ± 0.1 | -0.2 ± 0.1 |  |  |  |  |  |
| Mood swings | Placebo | -0.2 ± 0.1 | -0.3 ± 0.1 | -0.4 ± 0.1 | 0.9536 | 0.3488 | NA | NA | NA |
|  | aLA | -0.4 ± 0.1 | -0.4 ± 0.1 | -0.5 ± 0.1 |  |  |  |  |  |
| Depression | Placebo | -0.1 ± 0.1 | -0.2 ± 0.1 | -0.1 ± 0.1 | 0.5749 | 0.2446 | NA | NA | NA |
|  | aLA | -0.3 ± 0.1 | -0.3 ± 0.1 | -0.4 ± 0.1 |  |  |  |  |  |
| Decreased efficiency | Placebo | -0.2 ± 0.1 | -0.2 ± 0.1 | -0.2 ± 0.1 | 0.3328 | 0.1663 | NA | NA | NA |
|  | aLA | -0.5 ± 0.1 | -0.4 ± 0.1 | -0.3 ± 0.1 |  |  |  |  |  |
| Lowered motor  coordination | Placebo | 0 ± 0.1 | -0.1 ± 0.1 | -0.2 ± 0.1 | 0.1142 | **0.0250** | NA | NA | NA |
|  | aLA | -0.5 ± 0.1 | -0.3 ± 0.1 | -0.4 ± 0.1 |  |  |  |  |  |
| Tension | Placebo | -0.1 ± 0.1 | 0.0 ± 0.1 | 0.1 ± 0.1 | 0.9097 | 0.7837 | NA | NA | NA |
|  | aLA | 0.0 ± 0.1 | 0.1 ± 0.1 | 0.1 ± 0.1 |  |  |  |  |  |

Values are presented as LSM±SE. MMRM analyses were conducted. Group comparisons at each time point were evaluated when the group by time interaction effect was significant. aLA, alpha-lactalbumin; MDQ, menstrual distress questionnaire; MMRM, mixed-effects models with repeated measures; NA, not assessed.

**Table S3.** Changes of MDQ subscale scores during the menstrual phase.

|  |  | **LSM ± SE** | | | **MMRM, *P* value** | | | | |
| --- | --- | --- | --- | --- | --- | --- | --- | --- | --- |
| **Item** | **Group** | **Cycle 1** | **Cycle 2** | **Cycle 3** | **Group×Time interaction** | **Group main effect** | **Cycle 1** | **Cycle 2** | **Cycle 3** |
| Weight gain | Placebo | 0.0 ± 0.1 | 0.1 ± 0.1 | -0.1 ± 0.1 | 0.9744 | 0.2195 | NA | NA | NA |
|  | aLA | -0.2 ± 0.1 | -0.1 ± 0.1 | -0.2 ± 0.1 |  |  |  |  |  |
| Insomnia | Placebo | 0.0 ± 0.1 | 0.2 ± 0.1 | -0.1 ± 0.1 | 0.2299 | 0.2671 | NA | NA | NA |
|  | aLA | 0.1 ± 0.1 | 0.2 ± 0.1 | 0.2 ± 0.1 |  |  |  |  |  |
| Crying | Placebo | -0.1 ± 0.1 | 0.1 ± 0.1 | 0.0 ± 0.1 | 0.2304 | 0.1414 | NA | NA | NA |
|  | aLA | 0.1 ± 0.1 | 0.1 ± 0.1 | 0.2 ± 0.1 |  |  |  |  |  |
| Reduced school or  work performance | Placebo | -0.2 ± 0.1 | -0.1 ± 0.1 | 0.0 ± 0.1 | 0.4392 | 0.9815 | NA | NA | NA |
|  | aLA | -0.1 ± 0.1 | -0.1 ± 0.1 | -0.2 ± 0.1 |  |  |  |  |  |
| Muscle stiffness | Placebo | -0.1 ± 0.1 | 0 ± 0.1 | -0.4 ± 0.1 | 0.0826 | 0.3479 | NA | NA | NA |
|  | aLA | -0.1 ± 0.1 | -0.4 ± 0.1 | -0.4 ± 0.1 |  |  |  |  |  |
| Forgetfulness | Placebo | 0.1 ± 0.1 | 0.0 ± 0.1 | 0.2 ± 0.1 | 0.0980 | 0.4312 | NA | NA | NA |
|  | aLA | -0.1 ± 0.1 | 0.1 ± 0.1 | 0.0 ± 0.1 |  |  |  |  |  |
| Confusion | Placebo | -0.1 ± 0.1 | 0.0 ± 0.1 | 0.0 ± 0.1 | 0.3466 | 0.7686 | NA | NA | NA |
|  | aLA | 0.0 ± 0.1 | 0.0 ± 0.1 | -0.2 ± 0.1 |  |  |  |  |  |
| Take naps;  stay in bed | Placebo | -0.2 ± 0.1 | -0.2 ± 0.1 | -0.4 ± 0.1 | 0.2095 | 0.8777 | NA | NA | NA |
|  | aLA | -0.2 ± 0.1 | -0.4 ± 0.1 | -0.3 ± 0.1 |  |  |  |  |  |
| Headache | Placebo | 0.1 ± 0.1 | 0.0 ± 0.1 | 0.0 ± 0.1 | 0.4702 | 0.5952 | NA | NA | NA |
|  | aLA | 0.1 ± 0.1 | -0.2 ± 0.1 | 0.0 ± 0.1 |  |  |  |  |  |
| Skin disorders | Placebo | 0.2 ± 0.1 | 0.1 ± 0.1 | -0.2 ± 0.1 | 0.0586 | 0.5762 | NA | NA | NA |
|  | aLA | 0.0 ± 0.1 | -0.1 ± 0.1 | -0.1 ± 0.1 |  |  |  |  |  |
| Loneliness | Placebo | -0.1 ± 0.1 | -0.2 ± 0.1 | -0.1 ± 0.1 | 0.7292 | 0.3515 | NA | NA | NA |
|  | aLA | 0.0 ± 0.1 | -0.1 ± 0.1 | 0.0 ± 0.1 |  |  |  |  |  |
| Stay at home | Placebo | -0.3 ± 0.1 | -0.2 ± 0.1 | -0.5 ± 0.1 | 0.2603 | 0.9796 | NA | NA | NA |
|  | aLA | -0.3 ± 0.1 | -0.3 ± 0.1 | -0.4 ± 0.1 |  |  |  |  |  |
| Lower abdominal  pain | Placebo | -0.2 ± 0.1 | -0.3 ± 0.1 | -0.3 ± 0.1 | 0.8074 | 0.6783 | NA | NA | NA |
|  | aLA | -0.1 ± 0.1 | -0.3 ± 0.1 | -0.3 ± 0.1 |  |  |  |  |  |
| Dizziness, faintness | Placebo | 0.1 ± 0.1 | 0.0 ± 0.1 | 0.0 ± 0.1 | 0.4802 | 0.2949 | NA | NA | NA |
|  | aLA | 0.1 ± 0.1 | 0.2 ± 0.1 | 0.2 ± 0.1 |  |  |  |  |  |
| Avoid social activities | Placebo | 0.0 ± 0.1 | -0.1 ± 0.1 | -0.1 ± 0.1 | 0.5895 | 0.7189 | NA | NA | NA |
|  | aLA | 0.0 ± 0.1 | 0.1 ± 0.1 | -0.1 ± 0.1 |  |  |  |  |  |
| Anxiety | Placebo | 0.0 ± 0.1 | 0.0 ± 0.1 | 0.0 ± 0.1 | 0.3784 | 0.6617 | NA | NA | NA |
|  | aLA | 0.2 ± 0.1 | 0.0 ± 0.1 | 0.0 ± 0.1 |  |  |  |  |  |
| Backache | Placebo | -0.1 ± 0.1 | -0.1 ± 0.1 | -0.2 ± 0.1 | 0.7738 | 0.5067 | NA | NA | NA |
|  | aLA | 0.0 ± 0.1 | 0.0 ± 0.1 | -0.2 ± 0.1 |  |  |  |  |  |
| Cold sweats | Placebo | 0.0 ± 0.1 | 0.0 ± 0.1 | 0.0 ± 0.1 | 0.8192 | 0.7965 | NA | NA | NA |
|  | aLA | 0.0 ± 0.1 | 0.0 ± 0.1 | 0.1 ± 0.1 |  |  |  |  |  |
| Decreased judgment | Placebo | 0.2 ± 0.1 | 0.0 ± 0.1 | 0.0 ± 0.1 | 0.0651 | 0.8912 | NA | NA | NA |
|  | aLA | 0.0 ± 0.1 | 0.1 ± 0.1 | 0.1 ± 0.1 |  |  |  |  |  |
| Fatigue | Placebo | -0.1 ± 0.1 | -0.2 ± 0.1 | -0.2 ± 0.1 | 0.6075 | 0.4805 | NA | NA | NA |
|  | aLA | -0.1 ± 0.1 | -0.3 ± 0.1 | -0.3 ± 0.1 |  |  |  |  |  |
| Nausea, vomiting | Placebo | 0.1 ± 0.1 | 0.0 ± 0.1 | 0.0 ± 0.1 | 0.0230 | 0.7761 | 0.0760 | 0.2144 | 0.8070 |
|  | aLA | -0.1 ± 0.1 | 0.1 ± 0.1 | 0.0 ± 0.1 |  |  |  |  |  |
| Restlessness | Placebo | 0.2 ± 0.1 | 0.1 ± 0.1 | 0.0 ± 0.1 | 0.8170 | 0.5025 | NA | NA | NA |
|  | aLA | 0.0 ± 0.1 | 0.0 ± 0.1 | 0.0 ± 0.1 |  |  |  |  |  |
| Hot flashes | Placebo | 0.0 ± 0.1 | 0.0 ± 0.1 | -0.1 ± 0.1 | 0.4306 | 0.6384 | NA | NA | NA |
|  | aLA | -0.1 ± 0.1 | -0.1 ± 0.1 | -0.1 ± 0.1 |  |  |  |  |  |
| Difficulty  concentrating | Placebo | 0.0 ± 0.1 | -0.2 ± 0.1 | -0.1 ± 0.1 | 0.3248 | 0.5397 | NA | NA | NA |
|  | aLA | -0.1 ± 0.1 | 0.0 ± 0.1 | 0.0 ± 0.1 |  |  |  |  |  |
| Painful breasts | Placebo | -0.1 ± 0.1 | -0.1 ± 0.1 | 0.0 ± 0.1 | 0.9557 | 0.9581 | NA | NA | NA |
|  | aLA | -0.1 ± 0.1 | -0.1 ± 0.1 | 0.0 ± 0.1 |  |  |  |  |  |
| Distractibility | Placebo | 0.2 ± 0.1 | 0.1 ± 0.1 | 0.0 ± 0.1 | 0.7160 | 0.7118 | NA | NA | NA |
|  | aLA | 0.1 ± 0.1 | 0.1 ± 0.1 | -0.1 ± 0.1 |  |  |  |  |  |
| Swelling | Placebo | -0.2 ± 0.1 | -0.2 ± 0.1 | -0.1 ± 0.1 | 0.5913 | 0.6069 | NA | NA | NA |
|  | aLA | -0.2 ± 0.1 | 0.0 ± 0.1 | -0.1 ± 0.1 |  |  |  |  |  |
| Accidents | Placebo | 0.2 ± 0.1 | 0.0 ± 0.1 | 0.0 ± 0.1 | 0.0246 | 0.8522 | 0.1367 | 0.3311 | 0.8094 |
|  | aLA | 0.0 ± 0.1 | 0.1 ± 0.1 | 0.0 ± 0.1 |  |  |  |  |  |
| Irritability | Placebo | 0.1 ± 0.1 | 0.0 ± 0.1 | 0.0 ± 0.1 | 0.2857 | 0.2222 | NA | NA | NA |
|  | aLA | -0.1 ± 0.1 | 0.0 ± 0.1 | -0.3 ± 0.1 |  |  |  |  |  |
| General aches  and pains | Placebo | 0.1 ± 0.1 | -0.1 ± 0.1 | 0.0 ± 0.1 | 0.7172 | 0.6531 | NA | NA | NA |
|  | aLA | 0.0 ± 0.1 | 0.0 ± 0.1 | -0.1 ± 0.1 |  |  |  |  |  |
| Mood swings | Placebo | 0.0 ± 0.1 | 0.0 ± 0.1 | 0.0 ± 0.1 | 0.6802 | 0.1068 | NA | NA | NA |
|  | aLA | -0.2 ± 0.1 | -0.2 ± 0.1 | -0.2 ± 0.1 |  |  |  |  |  |
| Depression | Placebo | -0.1 ± 0.1 | 0.0 ± 0.1 | 0.0 ± 0.1 | 0.2969 | 0.9260 | NA | NA | NA |
|  | aLA | 0.0 ± 0.1 | 0.0 ± 0.1 | -0.1 ± 0.1 |  |  |  |  |  |
| Decreased efficiency | Placebo | 0.0 ± 0.1 | -0.2 ± 0.1 | -0.3 ± 0.1 | 0.3633 | 0.4630 | NA | NA | NA |
|  | aLA | -0.2 ± 0.1 | -0.3 ± 0.1 | -0.3 ± 0.1 |  |  |  |  |  |
| Lowered motor  coordination | Placebo | -0.1 ± 0.1 | -0.3 ± 0.1 | -0.3 ± 0.1 | 0.8433 | 0.3871 | NA | NA | NA |
|  | aLA | -0.3 ± 0.1 | -0.3 ± 0.1 | -0.3 ± 0.1 |  |  |  |  |  |
| Tension | Placebo | 0.1 ± 0.1 | 0.2 ± 0.1 | 0.1 ± 0.1 | 0.6791 | 0.3976 | NA | NA | NA |
|  | aLA | 0.0 ± 0.1 | 0.2 ± 0.1 | 0.0 ± 0.1 |  |  |  |  |  |

Values are presented as LSM±SE. MMRM analyses were conducted. Group comparisons at each time point were evaluated when the group by time interaction effect was significant. aLA, alpha-lactalbumin; MDQ, menstrual distress questionnaire; MMRM, mixed-effects models with repeated measures; NA, not assessed.

**Table S4.** Changes of VAS scores during the premenstrual phase.

|  |  | **LSM ± SE** | | | **MMRM, *P* value** | | | | |
| --- | --- | --- | --- | --- | --- | --- | --- | --- | --- |
| **Factors** | **Group** | **Cycle 1** | **Cycle 2** | **Cycle 3** | **Group×Time interaction** | **Group main effect** | **Cycle 1** | **Cycle 2** | **Cycle 3** |
| Depressed mood | Placebo | -9.3 ± 3.2 | -7.3 ± 3.4 | -6.6 ± 3.3 | 0.1431 | 0.8735 | NA | NA | NA |
|  | aLA | -5.0 ± 3.2 | -4.4 ± 3.4 | -12.1 ± 3.4 |  |  |  |  |  |
| Anxious | Placebo | -2.9 ± 3.1 | -0.6 ± 3.3 | -7.3 ± 3.2 | 0.7425 | 0.7492 | NA | NA | NA |
|  | aLA | -3.6 ± 3.0 | -3.7 ± 3.3 | -7.1 ± 3.2 |  |  |  |  |  |
| Mood swings | Placebo | -12.5 ± 3.2 | -11.8 ± 3.5 | -13.7 ± 3.3 | 0.4551 | 0.8639 | NA | NA | NA |
|  | aLA | -9.0 ± 3.1 | -10.7 ± 3.5 | -16.2 ± 3.3 |  |  |  |  |  |
| Irritability | Placebo | -13.9 ± 3.2 | -11.4 ± 3.6 | -11.0 ± 3.3 | 0.3045 | 0.5521 | NA | NA | NA |
|  | aLA | -7.3 ± 3.1 | -9.8 ± 3.6 | -12.5 ± 3.4 |  |  |  |  |  |
| Decreased interest in activities | Placebo | -4.5 ± 3.4 | -9.3 ± 3.3 | -11.2 ± 3.6 | 0.8262 | 0.5408 | NA | NA | NA |
|  | aLA | -8.7 ± 3.3 | -10.3 ± 3.3 | -13.2 ± 3.7 |  |  |  |  |  |
| Difficulty concentrating | Placebo | -9.6 ± 3.0 | -10.6 ± 3.0 | -11.2 ± 3.2 | 0.6962 | 0.7373 | NA | NA | NA |
|  | aLA | -6.7 ± 3.0 | -9.0 ± 3.0 | -12.2 ± 3.3 |  |  |  |  |  |
| Easy fatigability | Placebo | -9.1 ± 3.1 | -14.6 ± 3.4 | -16.4 ± 3.6 | 0.8166 | 0.8589 | NA | NA | NA |
|  | aLA | -9.6 ± 3.0 | -13.7 ± 3.4 | -18.8 ± 3.7 |  |  |  |  |  |
| Overeating | Placebo | -4.9 ± 3.7 | -8.2 ± 3.5 | -12.1 ± 3.4 | 0.4618 | 0.6972 | NA | NA | NA |
|  | aLA | -10.4 ± 3.6 | -8.6 ± 3.5 | -10.7 ± 3.5 |  |  |  |  |  |
| Unable to sleep | Placebo | 7.3 ± 3.2 | 4.0 ± 3.2 | 1.4 ± 3.1 | 0.5152 | 0.6499 | NA | NA | NA |
|  | aLA | 5.9 ± 3.1 | 7.3 ± 3.2 | 4.5 ± 3.2 |  |  |  |  |  |
| Sleeping more | Placebo | -7.3 ± 3.1 | -7.9 ± 3.3 | -16.3 ± 3.1 | 0.1845 | 0.3493 | NA | NA | NA |
|  | aLA | -14.1 ± 3.0 | -12.8 ± 3.3 | -13.9 ± 3.2 |  |  |  |  |  |
| Out of control | Placebo | -5.8 ± 3.2 | -1.7 ± 3.2 | -5.5 ± 2.9 | 0.4063 | 0.8459 | NA | NA | NA |
|  | aLA | -2.8 ± 3.1 | -4.8 ± 3.2 | -7.5 ± 3.0 |  |  |  |  |  |
| Physical symptoms | Placebo | -12.9 ± 3.5 | -18.0 ± 3.6 | -17.5 ± 3.6 | 0.6048 | 0.5841 | NA | NA | NA |
|  | aLA | -11.9 ± 3.4 | -12.8 ± 3.6 | -17.5 ± 3.6 |  |  |  |  |  |

Values are presented as LSM±SE. MMRM analyses were conducted. Group comparisons at each time point were evaluated when the group by time interaction effect was significant. aLA, alpha-lactalbumin; MMRM, mixed-effects models with repeated measures; NA, not assessed.

**Table S5.** Changes of VAS scores during the menstrual phase.

|  |  | **LSM ± SE** | | | **MMRM, *P* value** | | | | |
| --- | --- | --- | --- | --- | --- | --- | --- | --- | --- |
| **Factors** | **Group** | **Cycle 1** | **Cycle 2** | **Cycle 3** | **Group×Time interaction** | **Group main effect** | **Cycle 1** | **Cycle 2** | **Cycle 3** |
| Lower  abdominal pain | Placebo | 4.0 ± 3.5 | 3.7 ± 3.7 | 4.9 ± 3.3 | 0.7750 | 0.6234 | NA | NA | NA |
|  | aLA | 4.1 ± 3.5 | 1.4 ± 3.6 | 1.2 ± 3.4 |  |  |  |  |  |
| Back pain | Placebo | 1.0 ± 3.3 | 0.8 ± 3.4 | -2.5 ± 3.2 | 0.9772 | 0.9784 | NA | NA | NA |
|  | aLA | 1.5 ± 3.3 | 0.6 ± 3.4 | -3.1 ± 3.2 |  |  |  |  |  |
| Headache | Placebo | 5.8 ± 3.6 | 1.2 ± 3.6 | 3.0 ± 3.3 | 0.9972 | 0.7102 | NA | NA | NA |
|  | aLA | 4.6 ± 3.5 | -0.4 ± 3.5 | 1.8 ± 3.4 |  |  |  |  |  |
| Muscle  discomfort | Placebo | 0.7 ± 2.9 | 2.0 ± 2.8 | 3.5 ± 2.8 | 0.6790 | 0.7500 | NA | NA | NA |
|  | aLA | 1.0 ± 2.8 | 1.7 ± 2.8 | 0.4 ± 2.8 |  |  |  |  |  |
| Fatigue | Placebo | 1.3 ± 3.0 | -0.1 ± 3.2 | -2.3 ± 3.3 | 0.6735 | 0.2456 | NA | NA | NA |
|  | aLA | -0.2 ± 3.0 | -5.4 ± 3.2 | -7.7 ± 3.3 |  |  |  |  |  |
| Irritability | Placebo | 2.6 ± 3.1 | 2.9 ± 3.0 | -0.4 ± 3.0 | 0.8095 | 0.1921 | NA | NA | NA |
|  | aLA | -2.0 ± 3.0 | -0.8 ± 3.0 | -6.5 ± 3.0 |  |  |  |  |  |
| Skin  irritation | Placebo | 7.5 ± 3.6 | -0.4 ± 3.4 | -4.5 ± 3.3 | 0.6869 | 0.6005 | NA | NA | NA |
|  | aLA | 2.5 ± 3.5 | -1.1 ± 3.4 | -4.9 ± 3.3 |  |  |  |  |  |
| Depression | Placebo | -2.2 ± 3.1 | -3.6 ± 2.9 | -1.9 ± 2.9 | 0.6795 | 0.7510 | NA | NA | NA |
|  | aLA | 1.0 ± 3.0 | -2.4 ± 2.9 | -3.2 ± 2.9 |  |  |  |  |  |
| Insomnia | Placebo | 2.9 ± 2.8 | 8.4 ± 3.3 | 0.7 ± 2.9 | 0.4388 | 0.7751 | NA | NA | NA |
|  | aLA | 4.0 ± 2.8 | 6.4 ± 3.2 | 4.5 ± 2.9 |  |  |  |  |  |
| Cannot  concentrate | Placebo | 2.8 ± 3.1 | 4.2 ± 2.9 | 3.1 ± 2.8 | 0.7728 | 0.4252 | NA | NA | NA |
|  | aLA | 2.0 ± 3.0 | 0.6 ± 2.8 | -0.6 ± 2.8 |  |  |  |  |  |

Values are presented as LSM±SE. MMRM analyses were conducted. Group comparisons at each time point were evaluated when the group by time interaction effect was significant. aLA, alpha-lactalbumin; MMRM, mixed-effects models with repeated measures; NA, not assessed.

**Table S6.** PGs and their metabolites in urine

|  |  | **Before consumption:** | **Before consumption:** | **After consumption:** |
| --- | --- | --- | --- | --- |
| **Item** | **Group** | **nonmenstrual phase** | **menstrual phase** | **menstrual phase** |
| PGE2 (ng/g Cr) | Placebo | 313 ± 17 | 312 ± 19 | 308 ± 20 |
|  | aLA | 315 ± 19 | 337 ± 21 | 328 ± 17 |
| PGF2α (ng/g Cr) | Placebo | 216 ± 11 | 210 ± 16 | 183 ± 12 |
|  | aLA | 252 ± 21 | 235 ± 16 | 194 ± 22 |
| PGE2 metabolite  (ng/g Cr) | Placebo | 366 ± 29 | 427 ± 37 | 577 ± 59 ^†^ |
|  | aLA | 363 ± 29 | 398 ± 32 | 528 ± 53 ^†^ |
| PGF2α metabolite  (ng/g Cr) | Placebo | 3773 ± 207 ^†^ | 4639 ± 356 | 4425 ± 326 |
|  | aLA | 4293 ± 283 ^†^ | 5644 ± 420 | 5391 ± 373 |

Values are presented as Mean±SE. Student's t-test was conducted. ^†^*P*<0.05, vs before consumption during the menstrual phase. PGE2, prostaglandin E2; PGF2α, prostaglandin F2α.

**Table S7.** AEs (details).

| **Classification** | | **Total** | | **Mild** | | **Moderate** | |
| --- | --- | --- | --- | --- | --- | --- | --- |
|  | **Name of adverse event** | **aLA** | **Placebo** | **aLA** | **Placebo** | **aLA** | **Placebo** |
| Gastrointestinal disorders | | 10 (14.7) | 19 (27.5) | 10 (14.7) | 18 (26.1) | 1 (1.5) | 1 (1.4) |
|  | nausea | 1 (1.5) | 1 (1.4) | 1 (1.5) | 1 (1.4) | 0 (0.0) | 0 (0.0) |
|  | gastroenteritis | 2 (2.9) | 7 (10.1) | 1 (1.5) | 7 (10.1) | 1 (1.5) | 0 (0.0) |
|  | stomachache | 3 (4.4) | 2 (2.9) | 3 (4.4) | 2 (2.9) | 0 (0.0) | 0 (0.0) |
|  | diarrhea | 1 (1.5) | 3 (4.3) | 1 (1.5) | 3 (4.3) | 0 (0.0) | 0 (0.0) |
|  | infectious gastroenteritis | 0 (0.0) | 1 (1.4) | 0 (0.0) | 1 (1.4) | 0 (0.0) | 0 (0.0) |
|  | stomatitis | 1 (1.5) | 0 (0.0) | 1 (1.5) | 0 (0.0) | 0 (0.0) | 0 (0.0) |
|  | vomiting | 1 (1.5) | 1 (1.4) | 1 (1.5) | 1 (1.4) | 0 (0.0) | 0 (0.0) |
|  | abdominal pain | 5 (7.4) | 8 (11.6) | 5 (7.4) | 7 (10.1) | 0 (0.0) | 1 (1.4) |
|  | feeling of bloating | 1 (1.5) | 2 (2.9) | 1 (1.5) | 2 (2.9) | 0 (0.0) | 0 (0.0) |
|  | constipation | 0 (0.0) | 1 (1.4) | 0 (0.0) | 1 (1.4) | 0 (0.0) | 0 (0.0) |
| General disorders | | 6 (8.8) | 1 (1.4) | 6 (8.8) | 1 (1.4) | 0 (0.0) | 1 (1.4) |
|  | swelling | 2 (2.9) | 0 (0.0) | 2 (2.9) | 0 (0.0) | 0 (0.0) | 0 (0.0) |
|  | burning | 1 (1.5) | 0 (0.0) | 1 (1.5) | 0 (0.0) | 0 (0.0) | 0 (0.0) |
|  | arthralgia | 1 (1.5) | 0 (0.0) | 1 (1.5) | 0 (0.0) | 0 (0.0) | 0 (0.0) |
|  | malaise | 1 (1.5) | 1 (1.4) | 1 (1.5) | 0 (0.0) | 0 (0.0) | 1 (1.4) |
|  | fever | 3 (4.4) | 0 (0.0) | 3 (4.4) | 0 (0.0) | 0 (0.0) | 0 (0.0) |
|  | slight fever | 0 (0.0) | 1 (1.4) | 0 (0.0) | 1 (1.4) | 0 (0.0) | 0 (0.0) |
| Infectious diseases | | 14 (20.6) | 6 (8.7) | 13 (19.1) | 5 (7.2) | 1 (1.5) | 1 (1.4) |
|  | influenza virus infection | 1 (1.5) | 0 (0.0) | 0 (0.0) | 0 (0.0) | 1 (1.5) | 0 (0.0) |
|  | Coronavirus disease | 0 (0.0) | 1 (1.4) | 0 (0.0) | 0 (0.0) | 0 (0.0) | 1 (1.4) |
|  | common cold | 12 (17.6) | 5 (7.2) | 12 (17.6) | 5 (7.2) | 0 (0.0) | 0 (0.0) |
|  | nasal congestion | 1 (1.5) | 0 (0.0) | 1 (1.5) | 0 (0.0) | 0 (0.0) | 0 (0.0) |
|  | tonsillitis | 1 (1.5) | 0 (0.0) | 1 (1.5) | 0 (0.0) | 0 (0.0) | 0 (0.0) |
| Infectious gastroenteritis | | 1 (1.5) | 0 (0.0) | 0 (0.0) | 0 (0.0) | 1 (1.5) | 0 (0.0) |
|  | viral gastroenteritis | 1 (1.5) | 0 (0.0) | 0 (0.0) | 0 (0.0) | 1 (1.5) | 0 (0.0) |
| Eye disorders | | 3 (4.4) | 0 (0.0) | 3 (4.4) | 0 (0.0) | 0 (0.0) | 0 (0.0) |
|  | eyestrain | 1 (1.5) | 0 (0.0) | 1 (1.5) | 0 (0.0) | 0 (0.0) | 0 (0.0) |
|  | eye pain | 1 (1.5) | 0 (0.0) | 1 (1.5) | 0 (0.0) | 0 (0.0) | 0 (0.0) |
|  | itching of the eyes | 1 (1.5) | 0 (0.0) | 1 (1.5) | 0 (0.0) | 0 (0.0) | 0 (0.0) |
| Musculoskeletal disorders | | 1 (1.5) | 3 (4.3) | 1 (1.5) | 3 (4.3) | 0 (0.0) | 0 (0.0) |
|  | lumbago | 1 (1.5) | 3 (4.3) | 1 (1.5) | 3 (4.3) | 0 (0.0) | 0 (0.0) |
| Blood and lymphatic system disorders | | 1 (1.5) | 0 (0.0) | 0 (0.0) | 0 (0.0) | 1 (1.5) | 0 (0.0) |
|  | anaemia | 1 (1.5) | 0 (0.0) | 0 (0.0) | 0 (0.0) | 1 (1.5) | 0 (0.0) |
| Respiratory disorders | | 4 (5.9) | 6 (8.7) | 4 (5.9) | 6 (8.7) | 0 (0.0) | 0 (0.0) |
|  | sore throat | 3 (4.4) | 6 (8.7) | 3 (4.4) | 6 (8.7) | 0 (0.0) | 0 (0.0) |
|  | cough | 1 (1.5) | 0 (0.0) | 1 (1.5) | 0 (0.0) | 0 (0.0) | 0 (0.0) |
|  | rhinitis | 1 (1.5) | 0 (0.0) | 1 (1.5) | 0 (0.0) | 0 (0.0) | 0 (0.0) |
| Injury | | 2 (2.9) | 1 (1.4) | 2 (2.9) | 1 (1.4) | 0 (0.0) | 0 (0.0) |
|  | laceration | 0 (0.0) | 1 (1.4) | 0 (0.0) | 1 (1.4) | 0 (0.0) | 0 (0.0) |
|  | bruise | 1 (1.5) | 0 (0.0) | 1 (1.5) | 0 (0.0) | 0 (0.0) | 0 (0.0) |
|  | cat bite | 1 (1.5) | 0 (0.0) | 1 (1.5) | 0 (0.0) | 0 (0.0) | 0 (0.0) |
| Nervous system disorder | | 11 (16.2) | 13 (18.8) | 11 (16.2) | 13 (18.8) | 0 (0.0) | 0 (0.0) |
|  | Headache | 11 (16.2) | 11 (15.9) | 11 (16.2) | 11 (15.9) | 0 (0.0) | 0 (0.0) |
|  | migraine | 0 (0.0) | 2 (2.9) | 0 (0.0) | 2 (2.9) | 0 (0.0) | 0 (0.0) |
|  | vertigo | 0 (0.0) | 1 (1.4) | 0 (0.0) | 1 (1.4) | 0 (0.0) | 0 (0.0) |
| Skin and subcutaneous tissue disorders | | 3 (4.4) | 1 (1.4) | 3 (4.4) | 1 (1.4) | 0 (0.0) | 0 (0.0) |
|  | pimple | 1 (1.5) | 0 (0.0) | 1 (1.5) | 0 (0.0) | 0 (0.0) | 0 (0.0) |
|  | skin pruritus | 0 (0.0) | 1 (1.4) | 0 (0.0) | 1 (1.4) | 0 (0.0) | 0 (0.0) |
|  | chronic urticaria | 1 (1.5) | 0 (0.0) | 1 (1.5) | 0 (0.0) | 0 (0.0) | 0 (0.0) |
|  | urticaria | 1 (1.5) | 0 (0.0) | 1 (1.5) | 0 (0.0) | 0 (0.0) | 0 (0.0) |
| Ovarian dysfunction | | 1 (1.5) | 0 (0.0) | 1 (1.5) | 0 (0.0) | 0 (0.0) | 0 (0.0) |
|  | polycystic ovary syndrome | 1 (1.5) | 0 (0.0) | 1 (1.5) | 0 (0.0) | 0 (0.0) | 0 (0.0) |

The subject numbers (and percentages) of AEs are presented. No severe AEs or AEs attributable to this study were observed. AE, adverse event; aLA, alpha-lactalbumin.
